# Anomalous electrocaloric behaviors in (anti)ferroelectrics: a mini-review

**DOI:** 10.3389/fchem.2024.1476273

**Published:** 2024-10-23

**Authors:** Feng Li, Chunchang Wang, Lei Shan

**Affiliations:** ^1^ Information Materials and Intelligent Sensing Laboratory of Anhui Province, Institutes of Physical Science and Information Technology, Anhui University, Hefei, China; ^2^ Leibniz International Joint Research Center of Materials Sciences of Anhui Province, Anhui University, Hefei, China; ^3^ Laboratory of Dielectric Functional Materials, School of Materials Science and Engineering, Anhui University, Hefei, China

**Keywords:** electrocaloric effect, anomalous ECE, relaxors, antiferroelectrics, solid-state cooling

## Abstract

Solid-state cooling, represented by the electrocaloric effect (ECE) in (anti)ferroelectric materials, has emerged as an alternative green refrigeration technology by virtue of its high efficiency and miniaturization and is expected to substitute conventional vapor-compression. Significant progress has been made in developing high-performance EC materials since its revival. However, anomalous EC behaviors are frequently observed, including asymmetric and negative EC profiles, and the physical mechanism behind this is still under debate. Its rationalization is of great importance since full utilization of anomalous EC behaviors could enhance EC strength and/or cooling capacity. This mini-review gives a brief overview of research advances in EC anomalies in (anti)ferroelectrics with the hope of provoking thought on the design of reconstructed refrigeration cycles and superior EC materials for application in solid-state cooling devices.

## 1 Introduction

**FIGURE 1:**
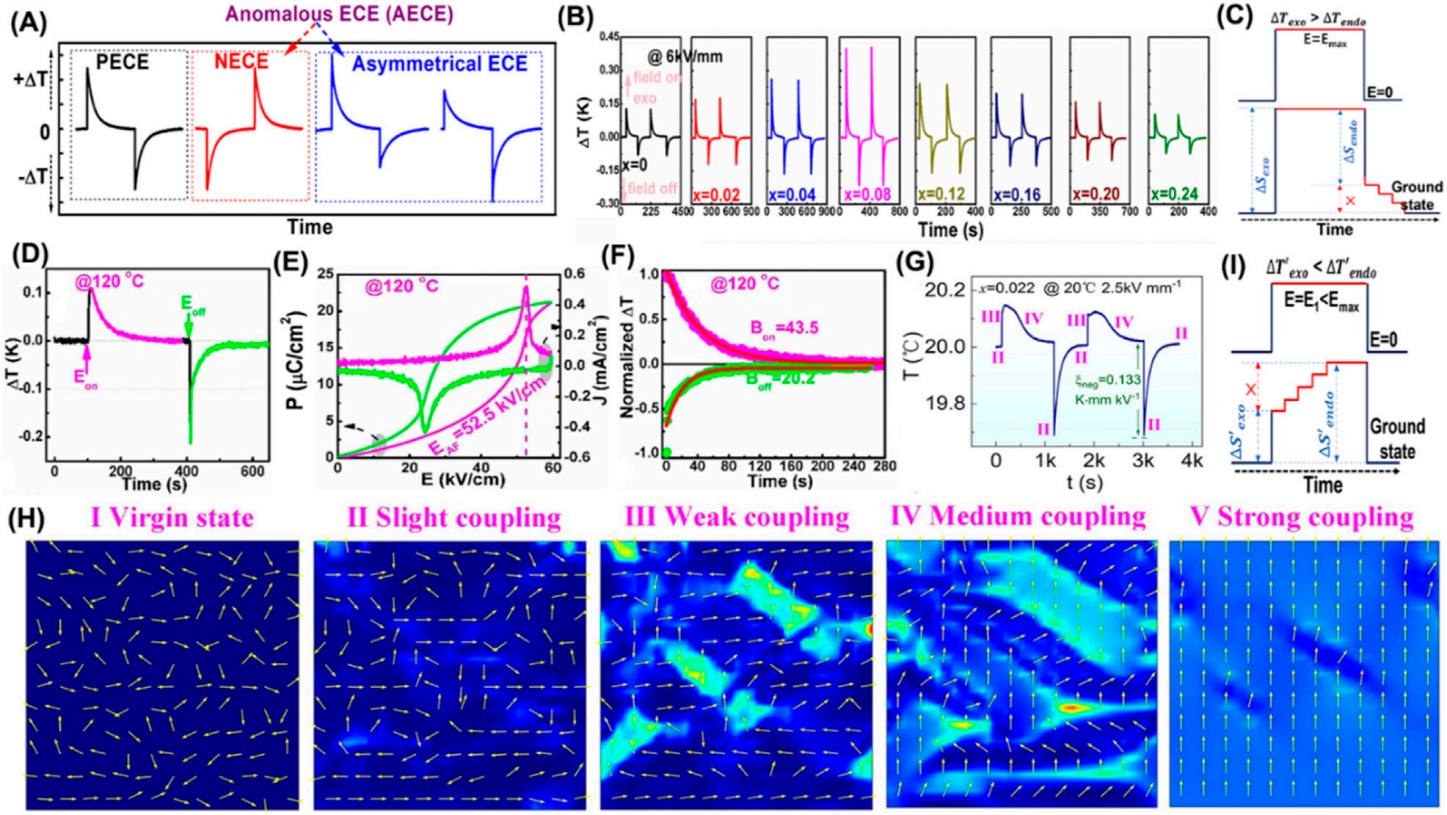
**(A)** Schematic profiles for positive ECE (PECE), negative ECE (NECE), and asymmetrical ECE. **(B)** EC profiles for poled Bi_0.5_Na_0.5_TiO_3_-BaTiO_3_-(Sr_0.7_Bi_0.2_□_0.1_)TiO_3_ (*x* = 0–0.24) ceramics; reproduced with permission from Li et al. (2017). **(C)** Schematic graph for ${∆S}_{exo}$ > ${∆S}_{endo}$ . **(D)** Asymmetric ECE. **(E)** Unipolar *P–E* and *J–E* loops at 120 °C. **(F)** Fitting results for exothermic and endothermic temperature signals for Pb [(Yb_1/2_Nb_1/2_)_0.84_(Mg_1/3_Nb_2/3_)_0.16_]O_3_ ceramic with high-order degree for *B*-site cations (denoted as PYMN); reproduced with permission from Li et al. (2023). **(G)** Asymmetric EC signal for (Bi_0.5_Na_0.45-*x*_K_0.05+*x*_)_0.92_Sr_0.08_Ti_0.99_Nb_0.01_O_3_ (*x* = 0.022) composition under *E* = 2.5 kV/mm at 20 °C; reproduced with permission from Li G. H. et al. (2022). **(H)** Different dipole coupling states in BNT-based relaxor ferroelectrics, where the background represents the elastic strain energy density; reproduced with permission from Li G. H. et al. (2022). **(I)** Schematic graph for ${∆S}_{exo}$ < ${∆S}_{endo}$ .

Conventional vapor compression technology has always occupied a dominant status in device cooling. However, it is being gradually phased out due to its global-warming potential and the difficulty of scaling it down to cool miniaturized chips (Shi et al., 2019; Valant, 2012). As a typical solid-state cooling method, ferroelectric cooling based on the electrocaloric effect (ECE) stands out because of its merits of high efficiency, easy integration, and miniaturization. ECE is defined as an entropy change with electric-field (*E*) stimuli in polar dielectrics; superior EC performances are largely explored in relaxors and antiferroelectrics (AFEs) contributed by relaxor–ferroelectric (FE) and AFE–FE phase transitions, respectively (Qian et al., 2023; Lu and Zhang, 2009). Admittedly, compared to sole ferroelectric domain reorientations, phase transitions with higher entropy change are expected to enable the design of excellent EC materials; for example, a giant temperature change *ΔT* ∼ 12 K is found in AFE PbZr_0.95_Ti_0.05_O_3_ thin films and relaxor copolymers P(VDF-TrFE-CFE) (Mischenko et al., 2006; Neese et al., 2008). Under normal circumstances, exothermic and endothermic peaks appear at nearly equal height as *E* is applied and released (positive ECE), and an inverse situation is defined for negative ECE (NECE) (Fan et al., 2022; Wu et al., 2022). Similarly, remarkable differences in height for exothermic and endothermic peaks are featured as asymmetrical EC profiles (Figure 1A). A question that currently concerns the ferroelectric cooling community is what is the physical mechanism behind for anomalous electrocaloric behaviors (AECE) in (anti)ferroelectric ceramics. Unfortunately, AEC behaviors have not been seriously considered before, and an understanding of it is essential not only for fundamental research but also for enhancing EC strength and/or cooling capacity. This is the focus in this mini-review. To characterize ECE, direct methods (e.g., temperature reading and heat flow measurement) and indirect methods with Maxwell relations are usually adopted (Molin et al., 2017; Birks et al., 2017; Lu et al., 2019). Here, direct-measured AECE and NECE properties in bulk ceramics are discussed.

## 2 Anomalous electrocaloric behaviors in (anti)ferroelectric ceramics

### 2.1 Asymmetrical EC profiles

Usually, exothermic and endothermic peaks appearing as *E* applied and released in normal EC materials and entropy/temperature change in dual processes (denoted ${∆T}_{exo}$ and ${∆T}_{endo}$ ) should be same. However, asymmetrical EC profiles are observed in Bi_0.5_Na_0.5_TiO_3_ (BNT)-based relaxors and Pb(Yb_0.5_Na_0.5_)O_3_-Pb(Mg_1/3_Nb_2/3_)O_3_ (PYMN) AFEs.

1) ${∆T}_{exo}$ > ${∆T}_{endo}$ in BNT-based ceramics under high *E* excitation, which is particularly remarkable in the coexisting region of nonergodic and ergodic relaxors (*x* = 0.08, Figure 1B) (Li et al., 2017). Mechanisms of electric hysteresis or Joule heat during domain switching have been proposed by Li J. J. et al. (2022) and Su et al. (2023). Recently, Wen et al. (2023) argued that a delayed polarization response is also an important factor since a recyclable remanent polarization/strain is observed as *E* is released (Wen et al., 2023). It is probably a time-dependent recovery behavior with slow relaxation dynamics (Figure 1C). In contrast, a larger ${∆T}_{endo}$ value is observed in the PYMN sample (Figure 1D), although a large *P–E* hysteresis is enclosed with high current density value (Figure 1E) (Li et al., 2023). A short relaxation time with low *B*_*off*_ value for endothermic curve is also fitted. This marks a faster relaxation dynamic and speed for the endothermic process and thus accounts for ${∆T}_{exo}$ < ${∆T}_{endo}$ (Figure 1F). Therefore, the above domain switching mechanism may not be a dominant factor for ${∆T}_{exo}$ > ${∆T}_{endo}$ in BNT-based ceramics, and relaxation dynamics play a leading role. This is associated with the intrinsic phase structure in BNT-based ceramics. It is notable that in both dominant NER (ferroelectric) or ER sides, this asymmetry is largely suppressed.
2) ${∆T}_{exo}$ < ${∆T}_{endo}$ in the low *E* range for BNT-based ceramics around the phase-coexistence region. Rationalization of this mechanism is crucial for boosting EC strength to realize a low *E*-driven cooling device. The ${∆T}_{endo}$ value surpasses that of ${∆T}_{exo}$ under low *E*, visualized by a diffuse and wide exothermic cusp and sharp endothermic peak (Figure 1G) (Li G. H. et al., 2022). Subsequently, diverse coupling states are presented to explain the underlying mechanism and underscore the evolution of distinct coupling states for justifying ${∆T}_{exo}$ < ${∆T}_{endo}$ (Figure 1H). In essence, a step-by-step II–III–IV process with *E* and a direct IV–II state without *E* results in a deeper endothermic profile. Coincidentally, this interesting phenomenon is also detected in the PYMN sample around the ferrielectric–AFE phase boundary (120 °C, Figure 1D). Ferrielectric is denoted as an intermediate state that displays a unique polarization configuration with either magnitude or angle modulation of dipoles instead of the strictly antiparallel one in typical AFEs (Liu et al., 2020). In fact, energy barriers are flattened, and mutual phase transitions are therefore facilitated in the phase coexistence region. Although *E* strength is not enough (or even absent) to instantly stimulate phase transitions, the coherent polar-units transition probably occurs during the isoelectric enthalpic transfer process (Figure 1I). This is reminiscent of the isothermal phase transition in BNT- and PbZrO_3_-based (anti)ferroelectrics, for which phase transition continuously advances in the proximity of phase boundaries with extending time (Li F. et al., 2022; Zhang et al., 2016). This may suggest an alternative mechanism for a deeper endothermic peak that a strong coupling state (FE) sluggishly grows out of the weak coupling (relaxor) parent matrix for time effect. This is also evidenced by a large amount of piezoelectricity developing at poling fields far below the coercive field in the BNT-BaTiO_3_ system, which is interpreted with the polarization alignment of *P4bm* polar nanodomains as time extends (Guo et al., 2013). Therefore, the time-dependent growth of the strong coupling (FE) phase accounts for ${∆T}_{exo}$ < ${∆T}_{endo}$ . In summary, time-dependent phase transition profoundly impacts EC behaviors and should be considered in EC analysis.

### 2.2 Negative ECE in AFE

**FIGURE 2:**
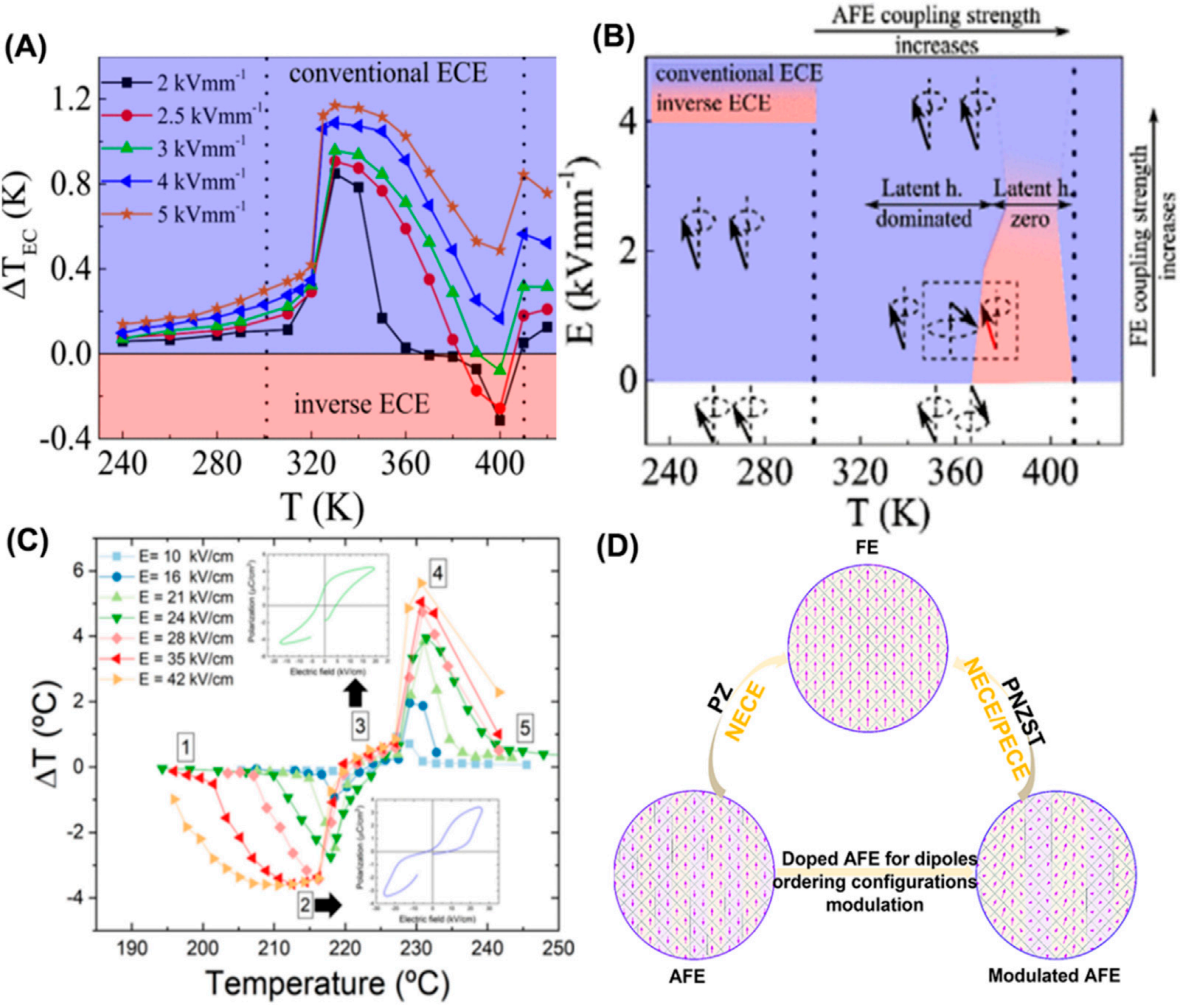
**(A)** PNZST ceramic features a crossover from positive to negative EC response with temperature and *E.* **(B)** Schematic representation of a possible mechanism responsible for EC behavior in PNZST; reproduced with permission from Novak et al. (2018). **(C)** *ΔT* of PbZrO_3_; the inset represents the *P-E* loops for different phases; reproduced with permission from Vales-Castro et al. (2021). **(D)** Dipoles ordering configurations in controlling EC behaviors in prototypical and modulated AFEs; adapted from Liu et al. (2020).

An insight into NECE is significant since a reorganized refrigeration cycle with the synergy of PECE and NECE is expected to improve the cooling efficiency. A giant NECE (*ΔT* ∼ −5 K) has been discovered in AFE La-doped Pb(Zr,Ti)O_3_ thin films, triggering research interest in NECE (Geng et al., 2015). The NECE is explained as an entropy increase by canting the dipoles under a moderate *E*. This mechanism could only account for NECE in a portion of AFEs, and instead it may be completely absent in other AFEs from low-to-high *E* sweep. Therefore, the underlying mechanism concerning NECE is still controversial. Novak et al. (2018) have determined that the EC property rests with temperature-dependent latent heat response and AFE coupling strength in Pb_0*.*99_Nb_0*.*02_ [(Zr_0*.*58_Sn_0*.*43_)_0*.*92_Ti_0*.*08_]_0*.*98_O_3_ (PNZST) ceramic (Figures 2A, B). A competitive role in AFE and FE strength, and latent heat finally produces an EC curve with an uncoordinated positive and negative EC evolution near separative FE–AFE and AFE–paraelectric (PE) phase transition regions, and NECE even disappears at high *E* across the AFE–PE phase boundary (Novak et al., 2018). Interestingly, a synergistic boosting of positive and negative *ΔT* is found in archetypal PbZrO_3_ AFE as *E* increases across AFE–FE and FE–PE phase transition (Figure 2C) instead of NECE extinction in PNZST ceramic. Vales-Castro et al. (2021) proposed an updated mechanism in which large NECE is based on endothermic AFE–FE switching instead of a main contribution from the dipole canting of the antiparallel lattice (Vales-Castro, et al., 2021). The similar EC behaviors are also found in *B*-site complex perovskite PbMg_0.5_W_0.5_O_3_ AFE, and a symmetric giant positive and negative *ΔT* appears at near room temperature (Li et al., 2021; Huang, et al., 2024).

Experimental evidence demonstrates that the pristine (rigid) and doped (soft) AFEs present distinct EC behavior. The stringent antiparallel arrangement of adjacent electric dipoles is established in prototypical PbZrO_3_ AFE, and the entropy change contributor solely stems from pure AFE–FE and PE–FE switching in AFE and PE regions. Therefore, a latent-heat-mediated ECE comes from the endothermic AFE–FE (*ΔT* = −3.5 K) and exothermic PE–FE phase transition (*ΔT* = + 5.6 K). Notably, both positive and negative *ΔT* grow as long as *E* increases before breakdown. However, it is entirely different in chemical modified PNZST ceramics. The dopants change the rigid AFE order and evolve into an intermediate ferrielectric (FiE) state with a flexible configuration and imbalanced polarization. Such an FiE with a competitive AFE and FE order diversely impacts EC properties with external stimuli of temperature and *E*. Generally, as temperature increases, 1) FE order switching facilitates (*ΔT* > 0), 2) and AFE coupling strength enhances and leads to an endothermic AFE–FE phase transition (*ΔT* < 0); 3) with a possible emergence of AFE/FE nanoclusters (*ΔT* > 0). Therefore, EC behavior undergoes a complex evolution with a superposition between AFE/FE coupling strength and latent heat contributions in FiE. This is also confirmed in a Pb(Yb_0.5_Nb_0.5_)O_3_-Pb(Mg_1/3_Nb_2/3_)O_3_ (PYMN) FiE sample with erratic EC behavior, of which a hop–hop character, asymmetric EC response, and NECE is simultaneously found as temperature evolves (Li et al., 2023). In addition, instantaneous endothermal behavior is observed in a Pb_0.97-*x*_Ba_*x*_La_0.02_Zr_0.95_Ti_0.05_O_3_ (*x* = 0.04) sample, further illustrating a complex thermal response in AFEs (Li et al., 2020). A remarkable EC difference between pure PZ and PNZST/PYMN FiE is that negative *ΔT* for the latter will be offset under high *E*, strongly indicating the competitive role of AFE and FE phases in controlling EC performances. Therefore, the above two mechanisms proposed by Novak et al. (2018) and Vales-Castro et al. (2021) are not mutually incompatible but complement each other. Dipoles ordering configurations in AFEs thus play a decisive role in EC properties and should be analyzed case-by-case (Figure 2D).

### 2.3 Artificially engineered NEC behaviors

**FIGURE 3:**
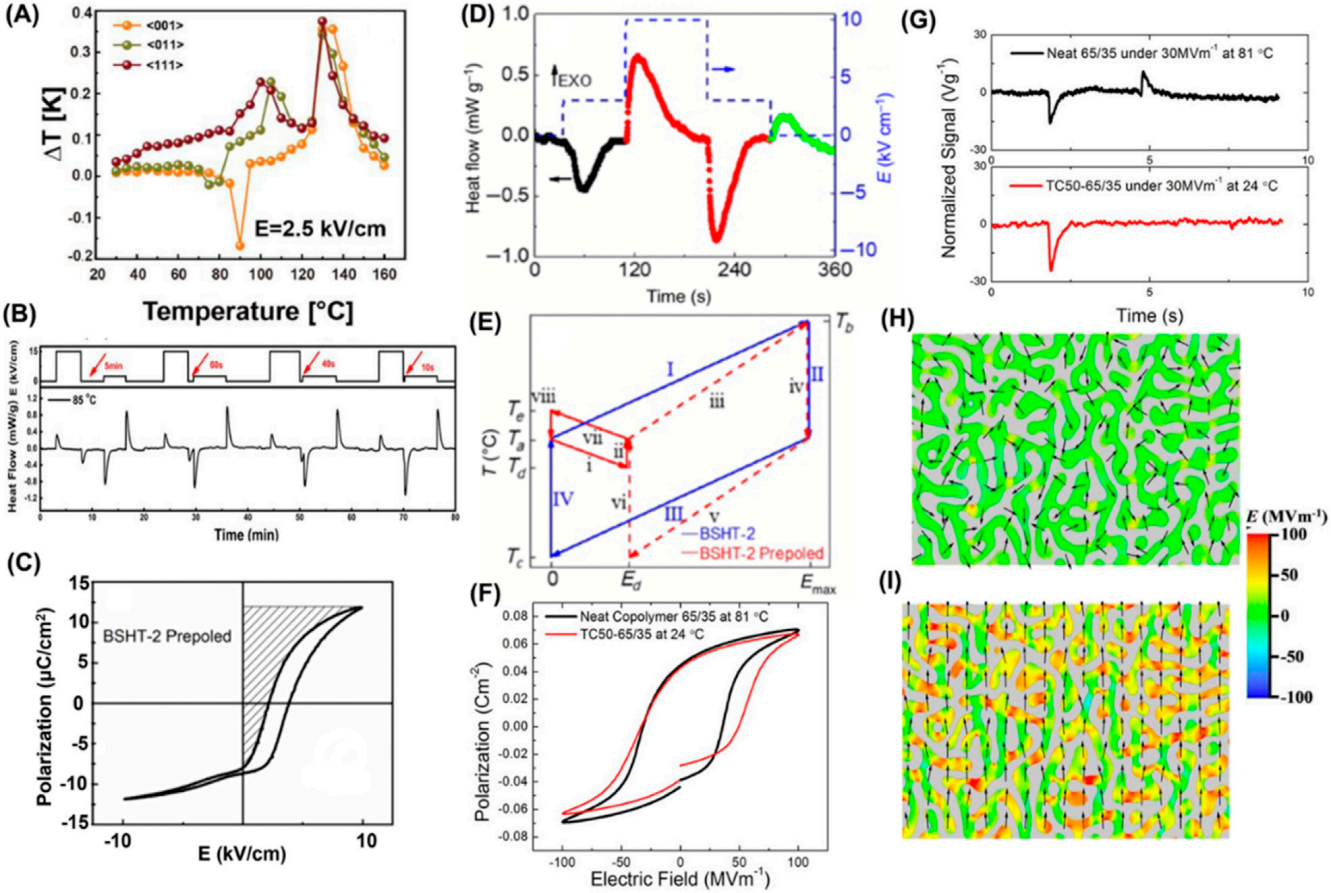
**(A)** Comparison of temperature-dependent *ΔT* for <001>-, <011>-, and <111>-oriented PMN-30PT single crystals; reproduced with permission from Li et al. (2020). **(B)** Alternate *E*_*1*_ = 15 and *E*_*2*_ = 2.5 kV/cm with varying interval time from 5 min to 10 s; reproduced with permission from Li et al. (2017). **(C)** Shifted *P*–*E* loop for pre-poled BSHT-2. **(D)** EC signal for poled BSHT-2. **(E)** Idealized refrigeration cycle utilizing both NECE and PECE based on a pre-poled BSHT-2 sample compared with conventional cycle; reproduced with permission from Li et al. (2021). **(F, G)** Comparison of *P*–*E* loops and heat-flux signals between TC50-65/35 blends and neat copolymer P(VDF-TrFE) 65/35 mol%. **(H, I)** Phase field simulation of dipolar direction distribution for TC50-65/35 blends in poled and de-poled states; reproduced with permission from Qian et al. (2016).

Except for AFEs, NECE can also be artificially engineered in FEs and relaxors. 1) The anisotropic (001)- and (011)-oriented 0.7Pb(Mg_1/3_Nb_2/3_)O_3_-0.3PbTiO_3_ (PMN-30PT) single crystal displays NECE under appropriate *E* and temperature; it originates from a monoclinic to tetragonal/orthogonal phase transition under noncollinear *E* (Figure 3A) (Li et al., 2020). Benefitting from the synergy of PECE and NECE in a (001)-oriented PMN-30PT single crystal at high (15 kV/cm) and low *E* (5 kV/cm), a significant 1.5× enhancement in cooling capacity is obtained by merging dual endothermic peaks (Figure 3B) (Li et al., 2017). Notably, this approach is easy to implement by simply adjusting interval time instead of a PE-to-AFE phase transition induced by the former EC cycle in AFEs (Li et al., 2020). 2) NECE can also be established by designing polar defects, such as in Ba_0.9_Sr_0.1_Hf_0.1_Ti_0.9_O_3_ ceramic. The pre-poled sample presents a ferro-restorable polarization feature capable of enhancing *ΔT* by up to 54% (Figure 3C). Moreover, both PECE and NECE emerge via a two-field step at *E*_*d*_ and *E*_*max*_ and enable a novel refrigeration cycle (Figures 3D, E). Therefore, the defect–dipole strategy is an elegant way to tailor EC performance in ferroelectrics (Li et al., 2021). Monte Carlo simulations also underscore the influence of defect dipoles on ECE in acceptor-doped BaTiO_3_ and reveal that in the case of antiparallel defect dipoles, the ECE can be positive or negative depending on the dipole density (Ma et al., 2016). 3) A hybrid normal ferroelectric/relaxor ferroelectric polymer blend is designed to obtain large cooling with an exclusion of a heating effect (Figures 3F, G); such a cooling response facilitates on-chip hotspot cooling. It is notable that this exotic EC response cannot occur in a sole neat copolymer and underlines the critical role of the relaxor end-member. This special EC response originates from the mesoscale dipolar interactions between ferroelectric/relaxor components, where dipole ordering in the poled relaxor polymer can be depolarized and stabilized with random distribution under a moderate inverse *E*, as simulated by a phase field (Figures 3H, I; Qian et al., 2016). The above artificial exotic EC behaviors may open many new application scenarios.

## 3 Concluding remarks

The electrocaloric effect is now a research frontier in solid-state cooling technology. The anomalous EC behaviors in (anti)ferroelectrics provide an alternative way of enhancing EC performance by realizing low-field high *ΔT* and engineering brand new refrigeration cycles. This mini-review provides an overview of research progress in asymmetrical EC profiles, negative EC, and engineered EC behaviors in bulk ceramics. However, the underlying physical mechanisms for these phenomena are still pending and deserve deeper exploration.

1) Although ${∆T}_{exo}$ < ${∆T}_{endo}$ contributes to a large EC strength and net endothermic effect in one cycle, the absolute ${∆T}_{endo}$ value is relatively low. The dipolar evolution in intermediate isoelectric enthalpic transfer is vague, spurring us to clarify the detailed mechanism by using *in situ* characterization, such as synchrotron XRD and Raman spectra. Another challenge is that, at present, this effect occurs in specific compositions (BNT-based relaxors and PYMN AFE) in a narrow temperature range. Exploring a series of such compositions within a wide temperature range is still tricky.
2) It is necessary when using a direct method to rigorously claim negative EC performance in (anti)ferroelectrics since the leakage and unsaturated polarization in *P–E* loops may interfere with calculation procedures using Maxwell’s relation (Lu et al., 2021). Large PEC and NEC values are obtained in pure PZ ceramic across high AFE–FE and FE–PE transition temperature. As the dopant is incorporated into PZ to move the transition toward room temperature, the modulated AFE state in PNZST results in competitive AFE and FE states (and/or AFE/FE nanoclusters), and EC is either positive or negative depending on *E* and temperature. This in fact impairs the absolute EC value, and how to fulfill a separated AFE/FE EC response in modulated AFEs is important. This will realize a better coupling of PECE and NECE to boost cooling capacity. The artificial EC behaviors also deserve attention since they provide an alternative approach to achieving NECE in FE and relaxors and are expected to open new application scenarios.
3) Intrinsically, EC behaviors are optimized in near-phase transition regions, such as FE–FE, AFE/FE–PE, and FE–relaxor. Additionally, enhancing breakdown strength in (anti)ferroelectrics is an extrinsic factor to enhanced ECE, such as thick-film ceramics (Wang et al., 2022). Recent novel avenues are supposed to improve the ECE. i) By utilizing temperature and electric field compensation mechanisms, the laminated BNT-based compositions with discrete *T*_*FR*_ are engineered (Lin et al., 2024). ii) Regulating the Schottky barrier at the grain boundary network in Ba_0.8_Zr_0.2_TiO_3_ ceramics and 2.4× enhancement of Δ*T* is achieved in annealed samples with a lower Schottky barrier (Xiao et al., 2024). Unfortunately, the design of large NEC materials seems to be elusive at present, and the method of improving PECE cannot be directly transferred to NECE. NECE may be even offset by PECE with increasing *E*, such as in soft PNZST AFE and <001>-PMN-PT single crystals; therefore, a critical *E* should be selected (Novak et al., 2018; Liao et al., 2024). It is thus promising though complex to artificially design high PECE and NECE in the future.
4) Theoretical models such as phase-field simulations and Monte Carlo (MC) simulations should be continuously optimized to predict and reveal series of high-performance EC materials, thus deepening recognition of the related physical mechanism (Hou et al., 2024; Fan et al., 2022; Xu et al., 2024). In addition to widely explored inorganic oxide counterparts, newly discovered molecular AFE and organometallic perovskite exhibit intriguing EC behaviors and reveal their great potential for solid-state refrigeration (Xu et al., 2021; Han et al., 2024). In summary, we hope that this mini-review serves as a catalyst for further development of high-performance EC materials and related physical mechanisms in the ferroelectric cooling community, laying a solid foundation for future practical applications.

## References

[B1] Birks E. Dunce M. Peräntie J. Hagberg J. Sternberg A. (2017). Direct and indirect determination of electrocaloric effect in Na_0.5_Bi_0.5_TiO_3_ . J. Appl. Phys. 121, 224102. 10.1063/1.4985067

[B2] Fan N. B. Íñiguez J. Bellaiche L. Xu B. (2022). Origin of negative electrocaloric effect in Pnma-type antiferroelectric perovskites. Phys. Rev. B 106, 224107. 10.1103/PhysRevB.106.224107

[B3] Geng W. P. Liu Y. Meng X. J. Bellaiche L. Scott J. F. Dkhil B. (2015). Giant negative electrocaloric effect in antiferroelectric La-doped Pb(ZrTi)O_3_ thin films near room temperature. Adv. Mater. 27, 3165–3169. 10.1002/adma.201501100 25864588

[B4] Guo H. Z. Ma C. Liu X. M. Tan X. L. (2013). Electrical poling below coercive field for large piezoelectricity. Appl. Phys. Lett. 102, 092902. 10.1063/1.4794866

[B5] Han S. G. Bie J. Fa W. Chen S. Tang L. W. Guo W. Q. (2024). Field-induced antiferroelectric-ferroelectric transformation in organometallic perovskite displaying giant negative electrocaloric effect. J. Am. Chem. Soc. 146, 8298–8307. 10.1021/jacs.3c13422 38498306

[B6] Hou X. Bin C. W. Zheng S. Z. Gao Z. G. Chen P. Wang J. (2024). Room temperature electrocaloric effect in PTO/STO superlattice induced by topological domain transition. Acta Mater 277, 120152. 10.1016/j.actamat.2024.120152

[B7] Huang Y. Y. Zhang L. Y. Ge P. J. Yang Y. L. Jing R. Y. Shur V. (2024). Boosting room-temperature electrocaloric performance in B-site complex antiferroelectrics: a synergistic design approach. Acta Mater 277, 120177. 10.1016/j.actamat.2024.120177

[B8] Li F. Chen G. Liu X. Zhai J. Shen B. Zeng H. R. (2017). Phase-composition and temperature dependence of electrocaloric effect in lead-free Bi_0.5_Na_0.5_TiO_3_-BaTiO_3_-(Sr_0.7_Bi_0.2_□_0.1_)TiO_3_ ceramics. J. Eur. Ceram. Soc. 37, 4732–4740. 10.1016/j.jeurceramsoc.2017.06.033

[B9] Li F. Liu W. Lou X. J. Zhai J. W. Wang C. C. (2022). Isothermal phase transition across phase boundary in (Pb_0.95_Ba_0.05_)ZrO_3_ ceramics. Appl. Phys. Lett. 120, 023902. 10.1063/5.0075892

[B10] Li F. Long M. S. Lou X. J. Wang C. C. Shan L. (2023). Order-degree-modulated ferroic response and an unconventional electrocaloric effect in B-site complex systems: a case study in Pb[(Yb_1/2_Nb_1/2_)_0.84_(Mg_1/3_Nb_2/3_)_0.16_]O_3_ ceramic. J. Phys. D. Appl. Phys. 56, 405501. 10.1088/1361-6463/ace1fe

[B11] Li G. H. Shi C. Zhu K. Ge G. L. Yan F. Lin J. F. (2022). Achieving synergistic electromechanical and electrocaloric responses by local structural evolution in lead-free BNT-based relaxor ferroelectrics. Chem. Eng. J. 431, 133386. 10.1016/j.cej.2021.133386

[B12] Li J. J. Li J. T. Wu H. H. Qin S. Q. Su X. P. Wang Y. (2020). Giant electrocaloric effect and ultrahigh refrigeration efficiency in antiferroelectric ceramics by morphotropic phase boundary design. ACS Appl. Mater. Interfaces 12, 45005–45014. 10.1021/acsami.0c13734 32924421

[B13] Li J. J. Su X. P. Wu H. H. Li J. T. Qin S. Q. Yin R. W. (2022). Electric hysteresis and validity of indirect electrocaloric characterization in antiferroelectric ceramics. Scr. Mater. 216, 114763. 10.1016/j.scriptamat.2022.114763

[B14] Li J. J. Wu H. H. Li J. T. Su X. P. Yin R. W. Qin S. Q. (2021). Room-temperature symmetric giant positive and negative electrocaloric effect in PbMg_0.5_W_0.5_O_3_ antiferroelectric ceramic. Adv. Funct. Mater. 33, 2101176. 10.1002/adfm.202101176

[B15] Li J. N. Lv J. Zhang D. W. Zhang L. X. Hao X. H. Wu M. (2021). Doping-induced polar defects improve the electrocaloric performance of Ba_0.9_Sr_0.1_Hf_0.1_Ti_0.9_O_3_ . Phys. Rev. Appl. 16, 014033. 10.1103/PhysRevApplied.16.014033

[B16] Li J. T. Qin S. Q. Bai Y. Li J. J. Qiao L. J. (2017). Flexible control of positive and negative electrocaloric effects under multiple fields for a giant improvement of cooling capacity. Appl. Phys. Lett. 111, 093901. 10.1063/1.4997068

[B17] Li J. T. Yin R. W. Su X. P. Wu H. H. Li J. J. Qin S. Q. (2020). Complex phase transitions and associated electrocaloric effects in different oriented PMN-30PT single crystals under multi-fields of electric field and temperature. Acta Mater 182, 250–256. 10.1016/j.actamat.2019.11.017

[B18] Liao L. C. Shan D. L. Lei C. H. Pan K. Li J. Y. Liu Y. Y. (2024). Revealing the mechanisms of electrocaloric effects by simultaneously direct measuring local electrocaloric and electrostrain under ambient conditions. Acta Mater 278, 120264. 10.1016/j.actamat.2024.120264

[B19] Lin W. K. Li G. H. Qian J. Ge G. L. Wang S. M. Lin J. F. (2024). Broadening the operating temperature span of the electrocaloric effect in lead-free ceramics via creating multi-stage phase transitions. J. Mater. Chem. A 12, 16438–16446. 10.1039/d4ta02319f

[B20] Liu H. Zhou Z. Y. Qiu Y. Gao B. T. Sun S. D. Lin K. (2020). An intriguing intermediate state as a bridge between antiferroelectric and ferroelectric perovskites. Mater. Horiz. 7, 1912–1918. 10.1039/D0MH00253D

[B21] Lu B. Yao Y. B. Jian X. D. Tao T. Liang B. Zhang Q. M. (2019). Enhancement of the electrocaloric effect over a wide temperature range in PLZT Ceramics by doping with Gd^3+^ and Sn^4+^ ions. J. Eur. Ceram. Soc. 39, 1093–1102. 10.1016/j.jeurceramsoc.2018.11.042

[B22] Lu S. G. Lin X. W. Li J. Li D. D. Yao Y. B. Tao T. (2021). Enhanced electrocaloric strengths at room temperature in (Sr_x_Ba_1-x_)(Sn_0.05_Ti_0.95_)O_3_ lead-free ceramics. J. Alloys Compd. 871, 159519. 10.1016/j.jallcom.2021.159519

[B23] Lu S. G. Zhang Q. M. (2009). Electrocaloric materials for solid-state refrigeration. Adv. Mater. 21, 1983–1987. 10.1002/adma.200802902

[B24] Ma Y. B. Grünebohm A. Meyer K. C. Albe K. Xu B. X. (2016). Positive and negative electrocaloric effect in BaTiO_3_ in the presence of defect dipoles. Phys. Rev. B 94, 094113. 10.1103/PhysRevB.94.094113

[B25] Mischenko A. S. Zhang Q. Scott J. F. Whatmore R. W. Mathur N. D. (2006). Giant electrocaloric effect in thin-film PbZr_0.95_Ti_0.05_O_3_ . Science 311, 1270–1271. 10.1126/science.1123811 16513978

[B26] Molin C. Peräntie J. Goupil F. L. Weyland F. Sanlialp M. Stingelin N. (2017). Comparison of direct electrocaloric characterization methods exemplified by 0.92Pb(Mg_1/3_Nb_2/3_)O_3_-0.08PbTiO_3_ multilayer ceramics. J. Am. Ceram. Soc. 100, 2885–2892. 10.1111/jace.14805

[B27] Neese B. Chu B. J. Lu S. G. Wang Y. Furman E. Zhang Q. M. (2008). Large electrocaloric effect in ferroelectric polymers near room temperature. Science 321, 821–823. 10.1126/science.1159655 18687960

[B28] Novak N. Weyland F. Patel S. Guo H. Z. Tan X. L. Rödel J. (2018). Interplay of conventional with inverse electrocaloric response in (Pb,Nb)(Zr,Sn,Ti)O_3_ antiferroelectric materials. Phys. Rev. B 97, 094113. 10.1103/PhysRevB.97.094113

[B29] Qian X. S. Chen X. Zhu L. Zhang Q. M. (2023). Fluoropolymer ferroelectrics: Multifunctional platform for polar-structured energy conversion. Science 380, eadg0902. 10.1126/science.adg0902 37167372

[B30] Qian X. S. Yang T. N. Zhang T. Chen L. Q. Zhang Q. M. (2016). Anomalous negative electrocaloric effect in a relaxor/normal ferroelectric polymer blend with controlled nano- and meso-dipolar couplings. Appl. Phys. Lett. 108, 142902. 10.1063/1.4944776

[B31] Shi J. Y. Han D. L. Li Z. C. Yang L. Lu S. G. Zhong Z. F. (2019). Electrocaloric cooling materials and devices for zero-global-warming-potential, high-efficiency refrigeration. Joule 3, 1200–1225. 10.1016/j.joule.2019.03.021

[B32] Su X. P. Li J. J. Hou Y. X. Yin R. W. Li J. T. Qin S. Q. (2023). Large electrocaloric effect over a wide temperature span in lead-free bismuth sodium titanate-based relaxor ferroelectrics. J. Materiomics 9, 289–298. 10.1016/j.jmat.2022.10.005

[B33] Valant M. (2012). Electrocaloric materials for future solid-state refrigeration technologies. Prog. Mater. Sci. 57, 980–1009. 10.1016/j.pmatsci.2012.02.001

[B34] Vales-Castro P. Faye R. Vellvehi M. Nouchokgwe Y. Perpiñà X. Caicedo J. M. (2021). Origin of large negative electrocaloric effect in antiferroelectric PbZrO_3_ . Phys. Rev. B 103, 054112. 10.1103/PhysRevB.103.054112

[B35] Wang S. B. Zhao P. F. Jian X. D. Yao Y. B. Tao T. Liang B. (2022). Large energy storage density and electrocaloric strength of Pb_0.97_La_0.02_(Zr_0.46-x_Sn_0.54_Ti_x_)O_3_ antiferroelectric thick film ceramics. Scr. Mater. 210, 114426. 10.1016/j.scriptamat.2021.114426

[B36] Wen L. J. Yin J. Wu X. J. Wei X. W. Liu W. B. Yang D. Y. (2023). Comprehending the underlying mechanism behind directly/indirectly obtained large electrocaloric response in Bi_0.5_Na_0.5_TiO_3_-based relaxor ferroelectrics. Acta Mater 255, 119090. 10.1016/j.actamat.2023.119090

[B37] Wu M. Xiao Y. N. Li H. Q. Liu Y. B. Gao J. H. Zhong L. S. (2022). Negative electrocaloric effects in antiferroelectric materials: a review. J. Inorg. Mater. 37, 376–386. 10.15541/jim20210420

[B38] Xiao W. R. Zhang C. Gong X. T. Qiu S. Y. Wang J. Y. Zhang H. B. (2024). Enhancement of electrocaloric effect in ferroelectric polycrystalline ceramics through grain boundary barrier engineering. Adv. Funct. Mater. 2405241. 10.1002/adfm.202405241

[B39] Xu H. J. Guo W. Q. Wang J. Q. Ma Y. Han S. G. Liu Y. (2021). A metal-free molecular antiferroelectric material showing high phase transition temperatures and large electrocaloric effects. J. Am. Chem. Soc. 143, 14379–14385. 10.1021/jacs.1c07521 34459600

[B40] Xu K. Shi X. M. Shao C. C. Dong S. Z. Huang H. B. (2024). Design of polar boundaries enhancing negative electrocaloric performance by antiferroelectric phase-field simulations. npj Comput. Mater. 10, 150. 10.1038/s41524-024-01334-2

[B41] Zhang D. W. Yao Y. G. Fang M. X. Luo Z. D. Zhang L. X. Li L. L. (2016). Isothermal phase transition and the transition temperature limitation in the lead-free (1-*x*)Bi_0.5_Na_0.5_TiO_3_-*x*BaTiO_3_ system. Acta Mater 103, 746–753. 10.1016/j.actamat.2015.10.037

